# Tai Chi is Effective in Delaying Cognitive Decline in Older Adults with Mild Cognitive Impairment: Evidence from a Systematic Review and Meta-Analysis

**DOI:** 10.1155/2020/3620534

**Published:** 2020-03-25

**Authors:** Jingjing Yang, Lulu Zhang, Qianyun Tang, Fengling Wang, Yu Li, Hua Peng, Shuhong Wang

**Affiliations:** ^1^Department of Nursing, Xiangya Hospital, Central South University, Changsha, Hunan 410008, China; ^2^Xiangya Nursing School, Central South University, Changsha, Hunan 410013, China

## Abstract

To determine whether Tai Chi (TC) is effective in slowing cognitive decline in older populations with mild cognitive impairment (MCI), we performed a systematic review and meta-analysis of randomized controlled trials (RCTs) on Tai Chi and MCI. We searched eight electronic databases (PubMed, PsycINFO, Wanfang, Web of Science, MEDLINE, CNKI, EBSCO, and the Cochrane Central Register of Controlled Trials) for appropriate RCTs published up to August 2019. For those studies included, the data were extracted, methodological quality was evaluated, and then meta-analysis was performed using Review Manager software (version 5.3). A total of 11 of the studies were available for systematic review, which together included 1061 participants, met the inclusion criteria, and ten of these were included in the meta-analysis. For most RCTs, the methodological quality was moderate. The meta-analysis revealed that Tai Chi could significantly improve global cognitive function; memory and learning; mental speed and attention; ideas, abstraction, figural creations, and mental flexibility; and visuospatial perception. The present review adds to the evidence showing that Tai Chi is potentially beneficial in improving cognitive functions among elderly people with MCI. However, strictly designed and well-reported RCTs are required.

## 1. Introduction

Mild cognitive impairment (MCI) is characterized by a deterioration in cognitive function, attention, memory, and learning that is beyond what would be expected considering age and educational level [1]. It is defined as the intermediate phase between the expected cognitive decline found in normal aging and the more serious decline found in early clinical dementia [2]. Nowadays, MCI has important implications for 10–20% of people over 65 years old [3], and there are approximately 2.5–7.5% of the population with MCI in the community [4, 5]. It is conservatively estimated that about 5–10% of patients with MCI develop dementia per year [6]. As this clinical cohort is associated with a particularly high risk of developing dementia, early interventions aiming to improve the cognitive status and stop further decline are the primary goal of MCI treatment. However, no pharmacological treatments are currently approved by the U.S. Food and Drug Administration for treating MCI or delaying the longer-term progression of MCI to dementia [7].

In recent decades, there has been increasing evidence to suggest that exercise should be considered a promising nonpharmacological intervention for improving cognition [8]. As an ancient Chinese martial art, Tai Chi has been gaining increasing popularity as an alternative form of physical exercise program in Western countries, such as America and England [9]. It is a type of psychophysiological exercise, based on the yin-yang theory of traditional Chinese medicine. And some studies indicated that Tai Chi could promote balance control, flexibility, muscular strength, and cardiorespiratory fitness for the practitioners, including children and adolescents [10–12]. Moreover, as it emphasizes mental concentration, physical balance, full-body stretching and relaxation, and relaxed breathing, Tai Chi has a great potential for becoming widely integrated into rehabilitation interventions for various medical and psychological conditions [13]. While a growing number of studies indicate that Tai Chi may improve cognitive function, for example, early meta-analyses suggested that Tai Chi could have potentially beneficial effects on the older adult such as preserving or improving the cognitive status and lowering the risk of dementia [14], in Yin Wu's meta-analysis, no relationship was shown between doing Tai Chi and the risk of cognitive decline in older adults [13]. Significantly, until the present study, there had been no systematic review and meta-analysis of randomized controlled trials (RCTs) to allow the efficacy of Tai Chi to be verified. So, it had been too early to make a definitive conclusion regarding the efficacy of Tai Chi on the cognitive deficits of MCI.

Therefore, in the current study, we performed a systematic review and meta-analysis of secondary data from RCTs to systematically evaluate the effectiveness of Tai Chi for cognitive functions among MCI patients as well as to determine if Tai Chi was useful for treating MCI.

## 2. Methods

Our study was performed and reported according to Preferred Reporting Items for Systematic Reviews and Meta-Analyses (PRISMA) [15].

### 2.1. Literature Search Strategy

We comprehensively searched PubMed, PsycINFO, Wanfang, Web of Science, MEDLINE, CNKI, EBSCO, and the Cochrane Central Register of Controlled Trials from inception up to August 28, 2019, with Chinese or English. The following search strings were used in the title/abstract/keywords/medical subject headings (MeSH): “Tai Chi” and “cognition” (or “memory” or “executive function” or “mild cognitive impairment” or “early-stage dementia” or “cognitive decline”). We also artificially searched the bibliography of the selected studies and previous reviews. Two researchers (L. Z. and N. Z.) independently performed the literature search.

### 2.2. Study Selection

We included those studies that fulfilled the following criteria, which were applied strictly. (1) *Participants.* The mean age was ≥60 years old, and they had a diagnosis of MCI (of any etiology) or Mini-Mental State Exam (MMSE) mean score 24–28 inclusive. Participants who had suffered head trauma, a stroke, or psychiatric disorders in the previous year were excluded. (2) *Intervention*. The duration of the experimental intervention was at least four weeks for Tai Chi, and the control group included no contact, waiting list, attention control, sham exercise, or alternative active treatment. However, studies combining Tai Chi with other interventions were eligible if the control group received the same parallel intervention. (3) *Study Design*. Only published RCTs on the effects of Tai Chi on MCI were included. (4) *Outcomes*. At least one measure for cognitive domains was reported so that an effect size could be calculated for cognitive performance or any specific cognitive domains measured by neuropsychological tests. Studies were excluded when the available information clearly indicated that the article was not eligible. Theses and short reports were also excluded. Any disagreements were resolved through a consensus meeting.

### 2.3. Data Extraction

Two authors (Q. T. and F. W.) independently extracted relevant data using a standardized form. The information extracted from eligible studies included first author, year of publication, participants' characteristics, sample size, study design, experimental setting and control intervention, duration, frequency, and all outcome measures. In addition, coding of outcomes into cognitive domains and effect direction was carried out independently by two reviewers (Q. T. and F. W.) in a manner similar to previous neuropsychological categorization published by Goodwill et al. [16] or in consultation with a senior reviewer (S. W.) (categorization of outcomes by domains, Table 1). Outcomes were recorded as mean and standard deviations (SDs) for each group at baseline and follow-up. For continuous outcomes, the sample size, mean, and standard deviation (SD) for each group at baseline and after intervention were extracted.

### 2.4. Assessment of Study Quality

The methodological quality of the studies that were included was assessed separately by two researchers (Q. T. and L. Z.) using the Physiotherapy Evidence Database (PEDro) scale with minor modification. As described previously [17], the revised PEDro scale consists of nine items (Table 2). Each item scored one point if the relevant information was explicitly presented, and there was a maximum score of nine per study. A PEDro score of six or more indicated high quality. A senior reviewer (S. W.) established consensus scores and resolved disagreements.

### 2.5. Statistical Analysis

Review Manager (RevMan) software (version 5.3) was utilized for the data analysis, and the statistical significance was defined as *p* value < 0.05. Continuous data were summarized using standardized mean differences (SMDs) with corresponding 95% confidence interval (CI). In addition, the magnitude of effect sizes (ESs) was calculated with Cohen's *d* and its 95% confidence intervals (CIs) and categorized as small (*d* ≥ 0.2), medium (*d* ≥ 0.5), or large (*d* ≥ 0.8) [18]. When studies provided more than one outcome per analysis domain, their mean difference and variance were combined into a single study-level estimate. Heterogeneity was examined using the *I*^2^ statistic. *I*^2^ ≤ 25% indicated low heterogeneity, *I*^2^ = 26–74% moderate, and *I*^2^ ≥ 75% high. The pooled effect was calculated using the random-effect model if heterogeneity was unsolvable.

## 3. Results

### 3.1. Literature Search

**Figure 1:**
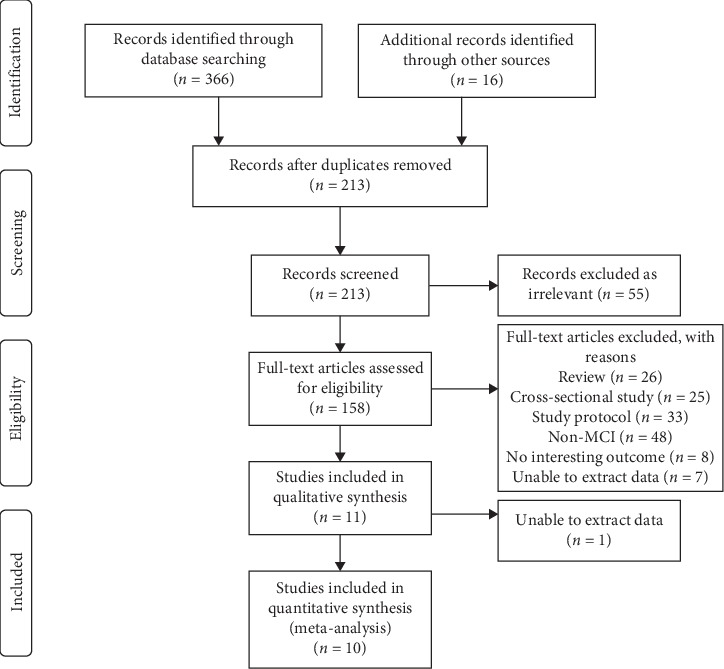
PRISMA flow diagram of study selection for systematic review and meta-analysis.

The process followed in the literature search and study selection is summarized in Figure 1. A total of 382 records were retrieved following the initial search. Two authors separately screened the records retrieved using the eligibility criteria. First, 169 duplicate articles were removed; next, another 51 obviously irrelevant records were also excluded. After this, 148 articles that included reviews (*n* = 26), cross-sectional studies (*n* = 31), study protocols (*n* = 33), non-MCI (*n* = 48), no interesting outcome (*n* = 8), or for which data could not be extracted (*n* = 7) were identified as not fulfilling the predetermined inclusion criteria. Finally, 11 available RCTs involving 1061 participants with MCI were considered for this meta-analysis [19–29]. Because the data from Fogarty et al. [25] were not available, 10 studies were finally procured for meta-analysis (Figure 1).

### 3.2. Study Characteristics

The 11 selected studies were published between 2011 and 2018 (Table 3). Collectively, 11 RCTs involving 1061 participants with MCI (mean age 74.1, range from 61.5 to 77.8 years) and a relatively high attrition rate (up to 24%) were included for review. Among these selected studies, six [19, 21, 23, 24, 27, 29] were carried out in China, and the other five in the USA [20], Vietnam [22], Thailand [26, 28], and England [25]. Interestingly, female participants made up 71.38%. Two studies [24, 29] did not report gender ratios. Five studies were based on 24-form Yang-style Tai Chi [19, 21, 22, 24, 29], and two studies were based on 10-form Yang-style Tai Chi [26, 28]. Four studies did not report which kind of Tai Chi was practiced [20, 23, 25, 27]. The duration of the Tai Chi training lasted from 10 weeks to 12 months, with the length of each session ranging from 30 to 120 minutes, while weekly frequency ranged from once a week to six times a week. Of these 11 studies, four compared Tai Chi with nonintervention (i.e., usual physical activity) [22, 23, 25, 30]. Although other studies compared Tai Chi training with stretching and toning exercises [19, 21], health education [20, 26, 28, 29], and playing cards or singing [24], we considered that those activities did not differ from nonintervention because they were of low intensity and did not significantly moderate the exercise habits of the participants. Adverse events did not occur during Tai Chi training across any of the selected studies.

### 3.3. Methodological Quality

The methodological quality of the studies that were included is displayed in Table 2. Quality scores range from five to nine points. Eleven trials (72.7%) were considered to be of high quality (PEDro score ≥ 6), with a mean value of 6.5 points. All studies get one point from eligibility criteria and between-group comparison. Seven [20, 23–26, 28, 29] studies reported more than 85% retention. One [23] trial failed to describe randomization.

Additionally, six studies [19, 21–23, 25, 27] lacked any mention of allocation concealment in reports. Other deducted points are related to the absence of blinded assessors [22–25, 27, 29], similar baseline [25], intent-to-treat analysis [23, 24, 27, 29], point measure, and measures of variability [25]. No studies were deemed fatally flawed.

### 3.4. Effect of Interventions

#### 3.4.1. Global Cognitive Function

**Figure 2:**
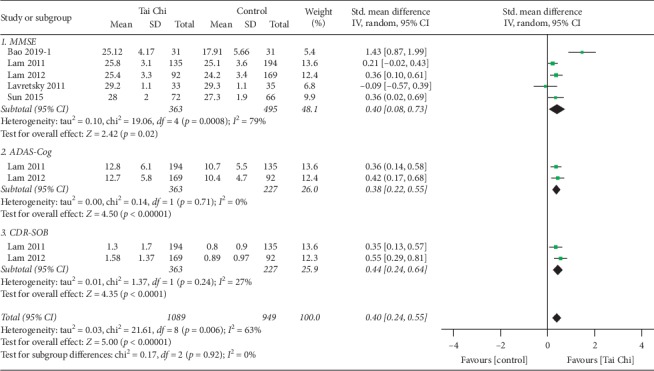
Forest plot of random-effects meta-analysis for Tai Chi on global cognitive ability.

Six studies explored the beneficial effects of Tai Chi training on global cognitive function in MCI patients assessed using MMSE [19–21, 24, 29], the Montreal Cognitive Assessment Scale (MoCA) [29], the Alzheimer's Disease Assessment Scale-Cognitive subscale (ADAS-Cog, in which scores are negatively correlated with global cognitive function) [19, 21], the Mattis Dementia Rating Scale (MDRS, in which scores are positively correlated with global cognitive function) [23], and the Clinical Dementia Rating Sum of Boxes (CDR-SOB) [19, 21]. Data were coded as a positive change score. Five studies investigated beneficial effects on global cognitive ability using MMSE, two studies [19, 21] using ADAS-Cog, and two studies [19, 21] using CDR-SOB. Given that MMSE is one of the most widely used scales for evaluating global cognitive function [31], we performed a subgroup analysis for MMSE, and the result suggested that Tai Chi training could significantly improve the global cognitive performance of MCI participants, as shown by significantly increased MMSE scores (*n* = 858, SMD = 0.40, 95% CI 0.08 to 0.73, *p*=0.0008, *I*^2^ = 79%; Figure 2). The corresponding effect size was medium (Cohen's *d* = 0.661, 95% CI −0.638 to 1.921). Previous research has shown that MMSE is less precise than CDR-SOB and ADAS-Cog in classifying the severity of cognitive dysfunction [32]. Therefore, to overcome the limitations of MMSE for MCI individuals, we also performed a subgroup analysis for ADAS-Cog scores (*n* = 590, SMD = 0.38, 95% CI 0.22 to 0.55, *p* < 0.00001, *I*^2^ = 0%, the random-effect model; Figure 2) and CDR-SOB scores (*n* = 590, SMD = 0.44, 95% CI 0.24 to 0.64, *p* < 0.0001, *I*^2^ = 27%, the random-effect model; Figure 2). The corresponding effect sizes for the ADAS-Cog scores and CDR-SOB were both small (Cohen's *d* = 0.397, 95% CI = −1.639 to 2.347 for ADAS-Cog scores, and Cohen's *d* = 0.468, 95% CI = −1.588 to 2.423 for CDR-SOB). In addition, the results of all measurements demonstrated that Tai Chi training did significantly improve global cognitive function as compared with the control groups (*n* = 858, SMD = 0.40, 95% CI 0.24 to 0.55, *p* < 0.00001, *I*^2^ = 63%, the random-effect model; Figure 2).

#### 3.4.2. Memory and Learning

**Figure 3:**
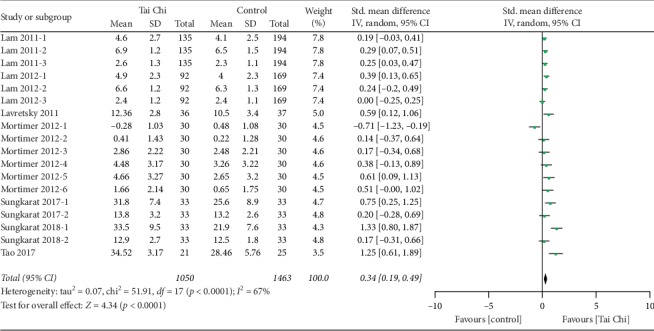
Forest plot of random-effects meta-analysis for Tai Chi on memory and learning ability.

Recent research suggests that cognitive tests focusing on an individual's memory and learning ability are most sensitive to MCI [33]. In this meta-analysis, seven studies, with a total of 855 participants, reported the effects of Tai Chi on memory and learning, as measured by delayed recall [19, 21], digit span (forward) [19, 21, 23], digit span (backward) [19, 21, 23, 26, 28], the California Verbal Learning Test [20], Rey Auditory Verbal Learning Test (immediate and delayed recall) [23], Mattis memory score [23], logical memory-delayed recall score [23], and Wechsler Memory Scale [30]. The result suggested that Tai Chi training could significantly improve the memory and learning scores of MCI patients compared with controls (*n* = 855, SMD = 0.37, 95% CI 0.24 to 0.51, *p* < 0.00001, *I*^2^ = 57%; Figure 3), corresponding to an effect size of 0.634 (Cohen's *d* = 0.634, 95% CI = −0.04 to 1.3).

#### 3.4.3. Mental Speed and Attention

**Figure 4:**
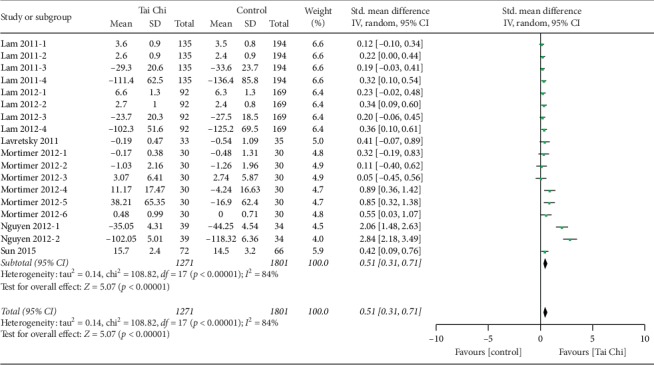
Forest plot of fixed-effects meta-analysis for Tai Chi on mental speed and attention.

In patients with MCI, mental speed and attention deficits appear at an early stage [34]. In this meta-analysis, six studies, which included 929 participants, reported the effects of Tai Chi training on mental speed and attention as measured by visual span (forward) [19, 21], visual span (backward) [19, 21], Stroop Color and Word Test [23], Mattis attention score and Frontal Assessment Battery (FAB) [24] (higher scores indicate better global cognitive function) and Chinese Trail A (seconds) [19, 21], Chinese Trail B (seconds) [19, 21], Trail-Making Test A (errors) [20], Stroop Color and Word Test [23], Trails A Time (seconds) [23], Trails B Time (seconds) [23], Trail-Making Test A (TMT Part A) [22], and Trail-Making Test B (TMT Part B) [22] (lower scores indicate better mental speed and attention). In order to make all quantitative data occur in the same direction, we coded these data as a positive change score when the lower scores indicated better mental speed and attention. Results revealed that MCI patients in Tai Chi training groups had a significant improvement in mental speed and attention scores compared with controls (*n* = 929, SMD = 0.51, 95% CI 0.31 to 0.71, *p* < 0.00001, *I*^2^ = 84%, the random-effect model; Figure 4).

#### 3.4.4. Ideas, Abstraction, Figural Creations, and Mental Flexibility

**Figure 5:**
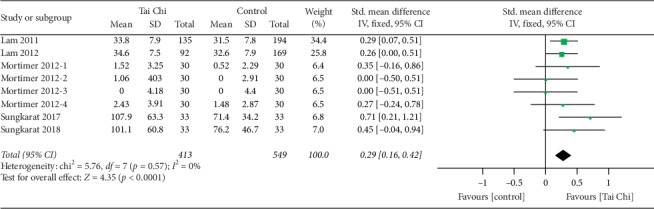
Forest plot of fixed-effects meta-analysis for Tai Chi on abilities related to ideas, abstraction, figural creations, and mental flexibility.

Five studies, with a total of 782 participants, reported the effects of Tai Chi on ideas, abstraction, figural creations, and mental flexibility as measured by category verbal fluency [19, 21], Mattis conceptualization score [23], Mattis initiation score [23], category verbal fluency (animals) [23], Wechsler Adult Intelligence Scale (WAIS) similarities [23], and Trail-Making Test Part B-A score [26, 28]. Because lower scores on the Trail-Making Test B-A indicate better performance, we coded these data as a positive change score. Results revealed that MCI patients in the Tai Chi training groups had a significant improvement in SMD scores related to ideas, abstraction, figural creations, and mental flexibility compared with controls (*n* = 782, SMD = 0.29, 95% CI 0.16 to 0.42, *p*=0.0001, *I*^2^ = 0%, the random-effect model; Figure 5), and the corresponding effect size was 0.172 (Cohen's *d* = 0.172, 95% CI = −0.813 to 1.151).

#### 3.4.5. Visuospatial Perception

**Figure 6:**
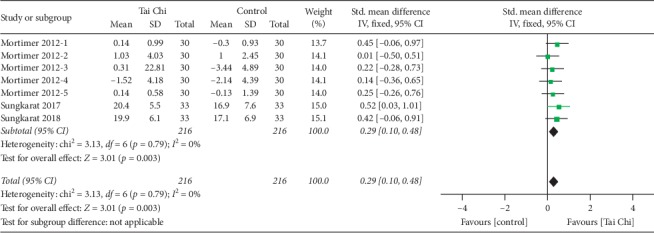
Forest plot of fixed-effects meta-analysis for Tai Chi on the ability of visuospatial perception.

Previously, reviews have shown that visuospatial perception (VSP) deficits can be detected in MCI [35]. Therefore, three studies, with a total of 192 participants, reported the effects of Tai Chi on visuospatial perception as measured by the Rey Figure Test (recall) [23], block design score [26, 28] (higher scores indicate better visuospatial perception function), clock drawing test [23], bell cancellation test [23], Mattis construction score [23], and Rey Figure Test (copying) (lower scores indicate better visuospatial perception function). Data were coded as a positive change score. Results showed that participants in the Tai Chi group showed a significant improvement in SMD scores related to visuospatial perception compared with controls (*n* = 192, SMD = 0.29, 95% CI 0.10 to 0.48, *p*=0.003, *I*^2^ = 0%, the random-effect model; Figure 6), and an effect size of 0.3 (Cohen's *d* = 0.3, 95% CI = −0.761 < *δ* < 1.348).

## 4. Discussion

### 4.1. Summary of Findings

We conduct, to the best of our knowledge, the first systematic review and meta-analysis of RCTs to objectively assess the effects of Tai Chi on cognitive performance among elderly people with MCI. In the end, a total of 10 RCTs, published between 2011 and 2018, were included in the meta-analysis. According to the *Cochrane Handbook for Systematic Reviews of Interventions*, the meta-analysis results reveal that Tai Chi can have moderate to significant benefits for global cognitive function (SMD = 0.35) in older adults with MCI, as suggested by previous studies [14]. Regarding the specific domain of cognition, Tai Chi may improve to a small to medium or significant degree memory and learning (SMD = 0.37), mental speed and attention (SMD = 0.51), ideas, abstraction, figural creations, and mental flexibility (SMD = 0.29), and visuospatial perception (SMD = 0.29).

### 4.2. The Potential Neurophysiological Mechanism by Which Tai Chi Benefits Cognitive Function

The neurophysiological mechanisms by which Tai Chi modulates cognitive processes are complex. Previous studies have suggested that there are some potential neurophysiological mechanisms inherent in Tai Chi training that may account for its beneficial effect on cognitive performance. For example, it was suggested that Tai Chi training was effective in increasing concentrations of plasma brain-derived neurotrophic factor (BDNF), a neurotrophin that is involved in neuron survival and plasticity critical for learning and memory [28]. In addition, Tai Chi training was found to be able to modulate the resting-state functional connectivity (rsFC) of the cognitive control network in the elderly, which can enhance rsFC between the hippocampus and medial prefrontal cortex, while decreasing bilateral dorsolateral prefrontal cortex rsFC with the left superior frontal gyrus and anterior cingulate cortex [27], which may play a crucial role in the assimilation and consolidation of memory function [30]. Additionally, Tai Chi was shown to trigger angiogenesis and reduce the inflammatory state of the brain, thus bringing about cognitive improvement [36]. Of course, the potential mechanism needs further investigation.

### 4.3. Strengths and Limitations

As with previous meta-analyses, the present meta-analysis has a number of strengths and limitations. Regarding its strengths, firstly, only RCTs were included in our review and meta-analysis, which implies that the studies that were included used prospective observation and had a rigorous study design. Secondly, the meta-analysis was employed to quantitatively measure the effects of Tai Chi on cognitive performance. Thus, the design of the study was methodologically stronger than those used in previous reviews [37]. In one review, only three RCTs were included, and no meta-analysis was conducted. Moreover, our meta-analysis adds to the qualitative review by Zheng et al. [37], since we were able to quantify the extent of the overall effect, confirming the efficacy of Tai Chi in MCI patients. In addition, previous meta-analyses focused on assessing the effects of Tai Chi training on cognitive function in elderly people with or without MCI and indicated that Tai Chi training could improve their cognitive function [38, 39]. In our study, we only paid attention to MCI in older adults in order to minimize potential sources of confounding bias and allow for generalist ability at a population level; those participants who suffered from cognitive impairment secondary to vascular dementia, or severe cognitive impairment due to degenerative dementia, were excluded. All of those strategies were favorable to supporting the demonstration of causal hypotheses related to Tai Chi and its outcomes.

Some potential limitations in the present review need to be acknowledged when analyzing and interpreting the current results. First, and perhaps foremost, the number of RCTs included and the sample size were unsatisfactory, although our review included 11 RCTs and over 1000 participants. More participants may yield more precise conclusions. For example, Barroso-Sousa et al. included 38 RCTs with 7551 patients to investigate the incidence and risk of endocrine adverse events following immune checkpoint inhibitor treatment [40]. Hunter et al. identified 37 studies comprising 53,891 participants to explore the association of social network interventions and health behavior outcomes [41]. Secondly, the methodological quality was also unsatisfactory for evaluating effects. For example, only five of the included trials reported allocation concealment in their experimental procedures, which may lead to selection bias or confounding of the final results. Moreover, performance bias may be inevitable, as it is impossible to blind the researchers and participants in the clinical experiment. Additionally, although we identified studies from sufficient databases, most of the trials included were carried out in Chinese regions, which may cause selection bias to a certain degree. In addition, although our results suggested that TC is effective for MCI in older adults, the effect size for different cognitive domains was heterogeneous, and therefore, the results need to be interpreted cautiously. Finally, on account of the insufficient number of trials for each outcome, there was no funnel plot analysis for the assessment of publication bias.

### 4.4. Clinical Implications for Future Studies

Our present meta-analysis indicated that Tai Chi produces low to moderate positive effects. Given that Tai Chi is easily accessible and easy-to-learn, it can be practiced in small groups with an instructor or individually at home. Meanwhile, it is a low-risk economic activity with many advantages in physical and psychological; we suggested that Tai Chi could implement it in the public health system to prevent cognitive decline for older adults with MCI. Nevertheless, future research is warranted. Although Tai Chi has its roots in traditional Chinese medicine theory, the frequency, duration, and mode of Tai Chi training varied considerably across studies. Thus, we suggest using a standardized Tai Chi training programs, including fixed frequency, duration, and mode, to better investigate the effect of Tai Chi on cognitive performance. Also, the criteria for recruiting participants should be more specific and more systematic so as to improve representativeness and avoid bias. Of course, appropriate controls are also needed. Furthermore, the assessment of cognitive outcomes should identify which cognitive domains would benefit most, and physiological outcomes including functional and structural neuroimaging and circulating biochemical markers such as BDNF, neuron-specific enolase (NSE), and S100*β* should be employed to investigate the physiological mechanisms. Moreover, researchers should report on their research according to the Consolidated Standards of Reporting Trials (CONSORT) guidelines in order to facilitate better quality evaluation.

## 5. Conclusion

In summary, our findings suggest that Tai Chi training can be effective in improving global cognitive function, as well as cognitive domains including memory and learning, mental speed and attention, visuospatial perception, language, and ideas, abstraction, and figural creations, among the elderly with MCI. As is evident from our review, Tai Chi should be considered a part of healthy lifestyle activities for the special population at a higher risk of developing dementia. However, given these methodological limitations, future rigorously designed studies will be required to strengthen the evidence for the cognitive benefits of Tai Chi training for MCI.

## Figures and Tables

**Table 1:** Neuropsychological test and cognitive abilities measured in each study.

Cognitive abilities	Neuropsychological test used in assessment
General cognition	Mini-Mental State Examination, Alzheimer's Disease Assessment Scale-Cognitive subscale (ADAS-Cog), Montreal Cognitive Assessment Scale (MoCA), Clinical Dementia Rating (CDR) Sum of Boxes, Mattis Dementing Rating Scale (total score)
Mental speed and attention	Stroop color-word, Trail-Making Test A and B, visual span Mattis attention score, Frontal Assessment Battery
Memory and learning	Ray Auditory Verbal Learning Test, California Verbal Learning Test, WAIS-Logical memory delay, Wechsler Memory Scale-recall, delayed recall, digit span-forward, digit span-back, Mattis memory score
Visuospatial perception	Rey-Osterrieth complex Figure-Copy, Clock drawing (copy and command), Block design Mattis construction score Bell cancellation test (visual-spatial function was assessed by tests of bell cancellation)
Ideas, abstraction, figural creations, and mental flexibility	WAIS similarities, Trail-Making Test (B-A), verbal fluency, Mattis conceptualization score, Mattis initiation score

**Table 2:** Study quality assessment of included studies.

Reference	Item 1	Item 2	Item 3	Item 4	Item 5	Item 6	Item 7	Item 8	Item 9	Sum score
Lam et al. 2011 [19]	1	1	0	1	1	0	1	1	1	7/9
Lavretsky et al. 2011 [20]	1	1	1	1	1	1	1	1	1	9/9
Lam et al., 2012 [21]	1	1	0	1	1	0	1	1	1	7/9
Nguyen et al., 2012 [22]	1	1	0	1	0	0	1	1	1	6/9
Mortimer et al., 2012 [23]	1	0	0	1	0	1	0	1	1	5/9
Sun et al., 2015 [24]	1	1	1	1	0	1	0	1	1	7/9
Fogarty et al., 2016 [25]	1	1	0	0	0	1	1	1	0	5/9
Sungkarat et al., 2017 [26]	1	1	1	1	1	1	1	1	1	9/9
Tao et al., 2017 [27]	1	1	0	1	0	0	0	1	1	5/9
Sungkarat et al., 2018 [28]	1	1	1	1	1	1	1	1	1	9/9
Bao et al., 2019 [29]	1	1	1	1	0	1	0	1	1	7/9

NOTE. Item 1, eligibility criteria; Item 2, randomization; Item 3, concealed allocation; Item 4, similar baseline; Item 5, blinding of assessors; Item 6, more than 85% retention; Item 7, missing data management (intent-to-treat analysis); Item 8, between-group comparison; Item 9, point measure and measures of variability; 1, explicitly described and present in detail; 0, absent, inadequately described, or unclear.

**Table 3:** Study characteristics of included studies.

Author, year	Participant *N* (AT); M/W	Mean age	Intervention	Frequency and duration of intervention (Tai Chi)	Group and/or home	Setting	Outcomes/measure
Lam et al., 2011 [19]	389 (15.4%) 92 M/297 W	77.2	TG: 24 forms simplified Tai Chi (*n* = 171) CG: Stretching and toning exercise (*n* = 218)	3 × 30 min/week for 5 months	Both	China	MMSE; ADAS-Cog; category verbal fluency, delay recall; digit span (forward), digit span (backward); visual span (forward); visual span (backward); Chinese Trail A (sec); Chinese Trail B (sec); CDR Sum of Boxes
Lavretsky et al., 2011 [20]	73 (6.8%) 28 M/45 W	70.6	TG: Escitalopram plus Tai Chi (*n* = 36) CG: Escitalopram plus health education (*n* = 37)	one 120-minute session/week for10 weeks	Group	American	MMSE; Trail-Making Test A errors; California Verbal Learning Test; Trail-Making Test B; Stroop test
Lam et al., 2012 [21]	389 (32.9%) 92 M/297 W	77.8	TG: 24 forms simplified Tai Chi (*n* = 171) CG: Stretching and toning exercise (*n* = 218)	3 × 30 min/week for 12 months	Both	China	MMSE; ADAS-Cog; category verbal fluency; delay recall; digit span (forward); digit span (backward); visual span (forward); visual span (backward); Chinese Trail A (sec); Chinese Trail B (sec); CDR Sum of Boxes
Nguyen et al., 2012 [22]	96(24.0%) 48 M/48 W	69	TG: 24-form style Tai Chi (*n* = 48) CG: maintain routine daily activities(*n* = 48)	2 × 60 min/week for 6 months	Group	Vietnam	Trail-Making Test A Trail-Making Test B
Mortimer et al., 2012 [23]	60 (10.8%) 20 M/40 W	67.8	TG: Tai Chi (*n* = 30) CG: no-intervention control (*n* = 30)	3 × 50 min/week for 40 weeks	Group	China	WAIS digit span (forward); WAIS digit span (backward); bell cancellation test; Rey Figure (copying); Rey Figure (recall); Stroop test (word). Stroop test (color); Stroop test (color-word); Auditory Verbal Learning Test (immediate. recall); Auditory Verbal Learning Test (delayed recall); Auditory Verbal Learning Test (delayed recognition); Category verbal fluency (animals); WAIS similarities; Trails A Time (seconds). Trails B Time (seconds); clock drawing test; Boston Naming Test (correct names); Mattis Dementing Rating Scale (total score); Mattis attention score; Mattis initiation score; Mattis construction score; Mattis conceptualization score; Mattis memory score
Sun et al., 2015 [24]	150 (8%) NR	69.2	TG: 24-form Yang-style Tai Chi (*n* = 72) CG: nonathletic activities (playing cards or singing) (*n* = 66)	2 × 60 min/week for 6 months	NR	China	MMSE Frontal Assessment Battery (FAB)
Sungkarat et al., 2017 [26]	66 (10.6) 9 M/57 W	67.9	TG: 10-form Tai Chi (*n* = 33) CG: educational information related to cognition (*n* = 33)	3-week center-based 12-week home-based Tai Chi (50 minutes per session, 3 times per week)	Both	Thailand	LM-delayed recall DS-forward/backward Block design Trail-Making Test Part B–A
Tao et al., 2017 [27]	61 (41.3%) 20 M/41 W	61.5	TG: Tai Chi (*n* = 21) Baduanjin (*n* = 15) CG: Control (*n* = 25)	a 60-minute practice session 5 days per week for 12 weeks	NR	China	Wechsler Memory Scale
Sungkarat et al., 2018 [28]	66(15.2%) 9 M/57 W	67.9	TG: 10-form Tai Chi (*n* = 33) CG: received educational material that covered information related to cognition (*n* = 33)	Practiced at home for 50 min/session, 3 times/week for 6 months	Both	Thailand	Logical memory-delayed recall Digit span-forward/backward Block design Trail-Making Test Part B–A
Bao et al., 2019 [29]	62 (0%) 29 M/33 W	NR	TG: 24-form Yang-style Tai Chi Tai Chi (*n* = 31) CG: educational (*n* = 31)	30 min/session, 2 times/week for 6 months	NR	China	MMSE MoCA
Fogarty et al., 2016 [25]	40 (0%) 19 M/21 W	72 y	TG: Tai Chi plus memory training CG: memory training	2 × 90 min for 22 weeks	Group	England	Hopkins verbal learning test; digit span and digit Symbol; Trail-Making Test (TMT) A and B; Rivermead Behavioral memory test; second Edition Everyday attention

Abbreviations: ADAS-Cog, Alzheimer's Disease Assessment Scale-Cognitive subscale; AT, attrition rate; CDR, Clinical Dementia Rating; CG, control group; DS, digit span; LM, logical memory; MMSE, Mini-Mental State Examination; MoCA, Montreal Cognitive Assessment Scale; TG, treatment group; M/W, men/women; NR, not reported; TG, treatment group.
